# Chronobiological perspectives: Association between meal timing and sleep quality

**DOI:** 10.1371/journal.pone.0308172

**Published:** 2024-08-01

**Authors:** Li-Ming Yan, Hai-Jun Li, Qi Fan, Yi-Dong Xue, Tao Wang

**Affiliations:** 1 Department of Gynecology, The Affiliated Hospital of Yan’an University, Yan’an, Shaanxi, China; 2 Department of Neurology, The Affiliated Hospital of Yan’an University, Yan’an, Shaanxi, China; Federal University of Rio Grande do Sul: Universidade Federal do Rio Grande do Sul, BRAZIL

## Abstract

**Background:**

Meal timing has been associated with metabolism and cardiovascular diseases; however, the relationship between meal timing and sleep quality remains inconclusive.

**Objective:**

This study aims to investigate the relationship between meal timing and sleep quality from a chronobiological perspective.

**Methods:**

This study utilized data from the NHANES for the years 2005–2008, including a cohort of 7,023 participants after applying exclusion criteria. Sleep quality was assessed using the Pittsburgh Sleep Quality Index (PSQI). Meal timing was analyzed based on two 24-hour dietary recalls from each individual, considering the timing of the initial and final meals, meal duration, and frequency of meal occasions. Multiple linear regression models and hierarchical analyses were employed to examine the relationship between meal timing and PSQI scores, adjusting for various demographic and habitat covariates.

**Results:**

Statistical analysis revealed a positive correlation between delayed meal timings, increased meal occasions, and elevated PSQI scores, indicating that later meal timing are intricately linked with diminished sleep quality. Both later meal timings and more frequent meal occasions were significantly associated with poorer sleep quality. Compared to the first tertile, the β (95%CI) values of the third tertile were 0.545 (0.226, 0.864) for first meal timing, 0.586 (0.277, 0.896) for midpoint meal timing, 0.385 (0.090, 0.680) for last meal timing, and 0.332 (0.021, 0.642) for meal occasions in the adjusted models.

**Conclusion:**

These findings suggest that late initial, midpoint, and final meal timing, as well as more frequent meal occasions, are chrono-nutrition patterns associated with poor sleep quality.

## Introduction

Sleep and eating are fundamental elements of life-supporting behaviors, intricately interwoven, and constitute essential activities for sustaining life and health [1]. In recent years, sleep disorders have emerged as a severe public health issue, confirmed to be a risk factor for numerous adverse health outcomes, including cardiovascular disease [2, 3], obesity and premature mortality [4].

Previous studies have reported that dietary quality, food types, energy intake, and dietary composition can affect sleep quality [5–7]. Recently, chrono-nutrition has emerged as a prominent field of study, investigating the interplay between food intake and circadian rhythms [8]. Most studies have concentrated on the effects of chrono-nutrition on metabolism [9]. However, it is increasingly recognized that sleep may serve as a mediator due to the bidirectional relationship between diet and sleep, as well as the critical role of sleep in health [10]. Previous studies have indicated that the timing of meals can affect lifestyle habits and various hormone levels, which in turn may influence sleep quality [11]. However, recent studies have yielded mixed results regarding the impact of meal timing on sleep. Several studies have demonstrated that time-restricted eating (TRE) does not affect sleep quality, as assessed by the Pittsburgh Sleep Quality Index (PSQI) [12]. One study reported that long-term TRE could improve sleep quality [13]. On the other hand, it was found that no associations were observed between meal timing and sleep quality in older men [14]. Conversely, Falkenberg et al. reported that a longer period between evening meal consumption and bedtime was associated with shorter sleep duration [15]. Nevertheless, studies on meal timing and sleep quality, particularly those evaluating representative samples, remain limited.

We aim to explore the relationship between meal timing and sleep quality through a chrono-nutrition approach. Through this research, we seek to determine whether time-related dietary behaviors can affect sleep quality.

## Materials and approaches

### Participants

The National Health and Nutrition Examination Survey (NHANES), chestrated by the National Center for Health Statistics (NCHS) as part of the Centers for Disease Control and Prevention (CDC), serves as a comprehensive survey encapsulating the diverse demographic of the United States. This survey specifically focuses on noninstitutionalized U.S. residents, endeavoring to amass a vast array of health and nutritional data. Participation in NHANES is contingent upon obtaining informed written consent from the participants, in strict compliance with the ethical standards sanctioned by the National Center for Health Statistics Research Ethics Review Board (NCHS ERB), pursuant to Protocol #2005–06 and its subsequent extension covering the 2005–2006 and 2007–2008 cycles.

In the 2005–2006 and 2007–2008 NHANES cycles, an initial cohort of 20,497 participants was established. From this group, exclusions were made for 8,706 minors (below 18 years), 422 pregnant individuals, and cases with incomplete data in critical demographics and health metrics such as sex, age, race, the Pittsburgh Sleep Quality Index (PSQI), and sleep duration. Additionally, profiles with fewer than two meal occasions and missing data on covariates were also excluded. Consequently, the analysis was conducted on a refined dataset comprising 7,023 participants. This analysis included the computation of various health indices, notably the PSQI, sleep duration, timing of meals (including first and last), the midpoint of meal, meal duration, and frequency of meal occasions.

### Assessment of sleep quality

PSQI has been meticulously developed to evaluate sleep quality and patterns among adolescents and adults. During the NHANES 2005–2008 cycles, participants were required to complete eight self-reported items for the assessment of PSQI. Sleep latency was quantified via two specific items: (a) “How long does it usually take you to fall asleep at bedtime (minutes)?”, and (b) “In the past month, how often did you have trouble falling asleep?”. Similarly, sleep disturbances were gauged using two distinct items: (a) “In the past month, how often did you wake up during the night and have trouble getting back to sleep?” and (b) “In the past month, how often did you wake up early in the morning and was unable to get back to sleep?”. Concurrently, daytime dysfunction was assessed through another set of two items: (a) “In the past month, how often did you feel unrested during the day, no matter how many hours of sleep you had?” and (b) “In the past month, how often did you feel excessively or overly sleepy during the day?”. Typically, the time taken to fall asleep is categorized into four brackets: ≤15 minutes (score of 0), 16–30 minutes (score of 1), 31–60 minutes (score of 2), and ≥60 minutes (score of 3); And possible answers to the other questions were as follows: never-0 time a month (score of 0), rarely-1 time a month (score of 1), sometimes-2 to 4 times a month (score of 2), often-5 to 15 times a month (score of 3), and almost always-16 to 30 times a month (score of 4). The cumulative PSQI score, ranging from 0 to 23, is derived by summing the individual scores for each component: latency, disturbances, and daytime dysfunction. A PSQI score below 5 signifies good sleep quality, whereas scores ranging from 5 to 10 denote moderate sleep quality. Scores exceeding 10 are indicative of poor sleep quality, with escalating scores reflecting progressively deteriorating sleep quality [16].

### Meal timing parameters

Meal timing data were meticulously gathered via individual two 24-hour dietary recalls conducted on non-consecutive days during both the 2005–2006 and 2007–2008 NHANES cycles. The initial dietary recall interviews were executed in-person by professionally trained interviewers specializing in dietary assessments. Subsequently, the second dietary recall was conducted telephonically, typically scheduled between 3 to 10 days following the first. In both NHANES cycles, the U.S. Department of Agriculture (USDA) Automated Multiple-Pass Method was employed which uses a 5-step procedure to quantify 24-h food and beverage intake [17]. Meal timing variables incorporated in this study included meal duration, timing of the first and last meals, midpoint of meal, and frequency of meal occasions. Meal duration was determined by calculating the interval in hours between the first and last meal of each participant, and the first meal episode was regarded as having occurred from 05:00. The midpoint of meal was ascertained by identifying the central time point between the first and last meal, i.e., ([Timing of the last meal—Timing of the first meal]/ 2) + Timing of the first meal. Meal occasions were quantified as the count of daily episodes involving the intake of food or beverages totaling 1 kcal or more at a single time point [18]. All analyzed variables represent the mean values derived from the two 24-hour dietary recalls.

### Covariates

Data extracted from the NHANES database encompassed an array of sociodemographic variables: age, gender, ethnicity, education level, poverty ratio, and marital status (classified as Married or living with partner; Widowed, Divorced or never married). Body Mass Index (BMI) data (kg/m^2^) were categorized as follows: Underweight (<18.5), Normal (18.5 to <25), Overweight (25 to <30), Obese (≥30). Smoking status was delineated into three groups: Never Smokers, Former Smokers, and Current Smokers; Drinking status was bifurcated into two categories: “Yes” for drinkers and “No” for non-drinkers; Sleep duration was recorded as a continuous variable, representing the total hours of sleep. The healthy eating index 2015 (HEI-2015) scores and total energy intake were calculated form two 24-hour dietary recalls and HEI-2015 was categorized as inadequate (less than 50), average (50 and 70) and optimal (more than 70) [19].

### Statistics

Data from various sources were amalgamated into a unified dataset in strict adherence to the NHANES protocol. Given our analysis’s emphasis on individual meal timing and considering the substantial individual variability and the multitude of influencing factors in sleep quality, our statistical approaches intentionally excluded weighted factors. Continuous variables were reported as mean ± standard deviation (SD), whereas categorical variables were delineated in terms of frequencies and percentages. Initially, participants were stratified into three distinct groups based on their PSQI scores: Good (<5), Moderate (5–10), and Poor (>10) [20]. Baseline characteristics were systematically summarized in accordance with the classification of PSQI scores. Group differences were evaluated using the F test for continuous variables and the chi-square test for categorical variables. Normality was assessed using the residual plot (Q-Q plot), homogeneity of variance was tested via the Breusch-Pagan test, and collinearity was examined by calculating the variance inflation factor (VIF). Identifying data that perfectly satisfy all the assumptions of linear regression is inherently challenging. Several studies [21–23] have encountered and addressed similar challenges using linear regression as their primary analytical method. Subsequently, a multiple linear regression model was employed to analyze the relationship between meal timing and PSQI scores. Multiple linear regression models were applied to adjust for various covariates, with results being presented across three analytical spectrums: unadjusted, minimally adjusted, and fully adjusted. In the unadjusted model, no covariates were considered. The minimally adjusted model (Model 1) incorporated age, gender, and race as its primary covariates. The fully adjusted model (Model 2) further included additional covariates: Education, Poverty ratio, Marital status, BMI, smoking status, Drinking habits, HEI-2015, and total energy intake. The selection of these covariates was grounded in findings from previous research investigations.

All statistical analyzes were performed using the R statistical software package (http://www.R-project.org, The R Foundation) P values less than 0.05 (two-sided) were considered statistically significant.

## Results

### Characteristics of the study population

Table 1 provides a comprehensive overview of the demographic characteristics of the participants. The study included a total of 7,023 participants, with a mean age of 50.54 ± 17.73 years. The study included a total of 7,023 participants, with a mean age of 50.54 ± 17.73 years. The majority of participants were Mexican American, comprising 52.5% of the total sample. The average initiation time for the first meal was recorded as 08:25. The median time for the second meal was observed to be 14:17. The average time for the final meal was recorded at 20:09. The average total duration of meal consumption was 12.16 hours. The average frequency of meals consumed was 4.70 times per day.

**Table 1:** Baseline characteristics of the study population.

Characteristic	Overall	Good	Moderate	Poor	P
	N = 7,023	N = 2,965	N = 2,339	N = 1,719	Value
**Age (years)**	50.54 (17.73)	51.46 (18.14)	50.06 (17.77)	49.62 (16.87)	**0.001**
**Sex**					**<0.001**
Male	3483 (49.6)	1657 (55.9)	1145 (49.0)	681 (39.6)	
Female	3540 (50.4)	1308 (44.1)	1194 (51.0)	1038 (60.4)	
**Race**					**<0.001**
Non-Hispanic White	1189 (16.9)	608 (20.5)	346 (14.8)	235 (13.7)	
Non-Hispanic Black	1433 (20.4)	665 (22.4)	448 (19.2)	320 (18.6)	
Mexican American	3685 (52.5)	1399 (47.2)	1308 (55.9)	978 (56.9)	
Other Hispanic	482 (6.9)	206 (6.9)	144 (6.2)	132 (7.7)	
Other/multiracial	234 (3.3)	87 (2.9)	93 (4.0)	54 (3.1)	
**Poverty Ratio**					**<0.001**
<1.3	1846 (26.3)	775 (26.1)	529 (22.6)	542 (31.5)	
1.3–3.5	2428 (34.6)	1021 (34.4)	892 (38.1)	515 (30.0)	
>3.5	2749 (39.1)	1169 (39.4)	918 (39.2)	662 (38.5)	
**Education**					**<0.001**
Less Than 9th Grade	1112 (15.8)	504 (17.0)	307 (13.1)	301 (17.5)	
9-11th Grade	1485 (21.1)	655 (22.1)	532 (22.7)	298 (17.3)	
High School Grad/GED	1707 (24.3)	652 (22.0)	602 (25.7)	453 (26.4)	
Some College or AA degree	755 (10.8)	367 (12.4)	210 (9.0)	178 (10.4)	
College Graduate or above	1964 (28.0)	787 (26.5)	688 (29.4)	489 (28.4)	
**PSQI**	7.14 (5.20)	2.36 (1.79)	7.87 (1.42)	14.40 (3.06)	**<0.001**
**Marital status**					**0.003**
Married or living with partner	4428 (63.0)	1904 (64.2)	1500 (64.1)	1024 (59.6)	
Widowed, divorced, never married	2595 (37.0)	1059 (35.8)	839 (35.8)	695 (40.4)	
**Smoke group**					**<0.001**
Current smoker	1499 (21.3)	555 (18.7)	474 (20.3)	470 (27.3)	
Former smoker	1889 (26.9)	818 (27.6)	628 (26.8)	443 (25.8)	
Never smoker	3635 (51.8)	1592 (53.7)	1237 (52.9)	806 (46.9)	
**Drinking**					**0.038**
No	2060 (29.4)	899 (30.3)	640 (27.4)	521 (30.3)	
Yes	4963 (70.7)	2066 (69.7)	1699 (72.6)	1198 (69.7)	
**Energy intake per day**					**<0.001**
Tertile 1	2341 (33.3)	1017 (34.3)	692 (29.6)	632 (36.8)	
Tertile 2	2341 (33.3)	963 (32.5)	817 (34.9)	561 (32.6)	
Tertile 3	2341 (33.3)	985 (33.2)	830 (35.5)	526 (30.6)	
**Healthy eating index 2015**					**0.002**
Inadequate	3551 (50.6)	1446 (48.8)	1169 (50.0)	936 (54.5)	
Average	2868 (40.8)	1240 (41.8)	969 (41.4)	659 (38.3)	
Optimal	604 (8.6)	279 (9.4)	201 (8.6)	124 (7.2)	
**Meal occasions**	4.70 (1.25)	4.63 (1.25)	4.76 (1.24)	4.73 (1.25)	**<0.001**
**Meal duration**	12.16 (2.28)	12.09 (2.20)	12.20 (2.28)	12.23 (2.44)	**0.007**
**First meal timing**	8:25(1:42)	8:23(1:42)	8:23(1:40)	8:31(1:44)	**0.017**
**Last meal timing**	20:09(1:29)	20:06(1:30)	20:11(1:27)	20:13(1:31)	**0.023**
**Meal midpoint**	14:17(1:15)	14:14(1:16)	14:17(1:14)	14:22(1:16)	**0.012**

PSQI, Pittsburgh Sleep Quality Index. Good, PSQI <5; Moderate, 5 = <PSQI = <10; Poor, PSQI>10.

In this study, 2,965 participants (42.22%) were categorized as having good sleep quality, 2,339 (33.30%) as having moderate sleep quality, and 1,719 (24.48%) as having poor sleep quality. In the group with good sleep quality, several notable characteristics were observed. This group tended to be older, had more males, fewer smokers, and exhibited higher dietary quality. Among those with good sleep quality, the average time for the first meal was 08:23, the midpoint of meal was at 14:14, and the last meal was at 20:06. The total duration of dietary intake was approximately 12.09 hours, with an average of 4.63 meals per day. Compared to participants with poor sleep quality, those with good sleep quality tended to have earlier first, middle, and last meal timings and fewer meal occasions.

**Fig 1:**
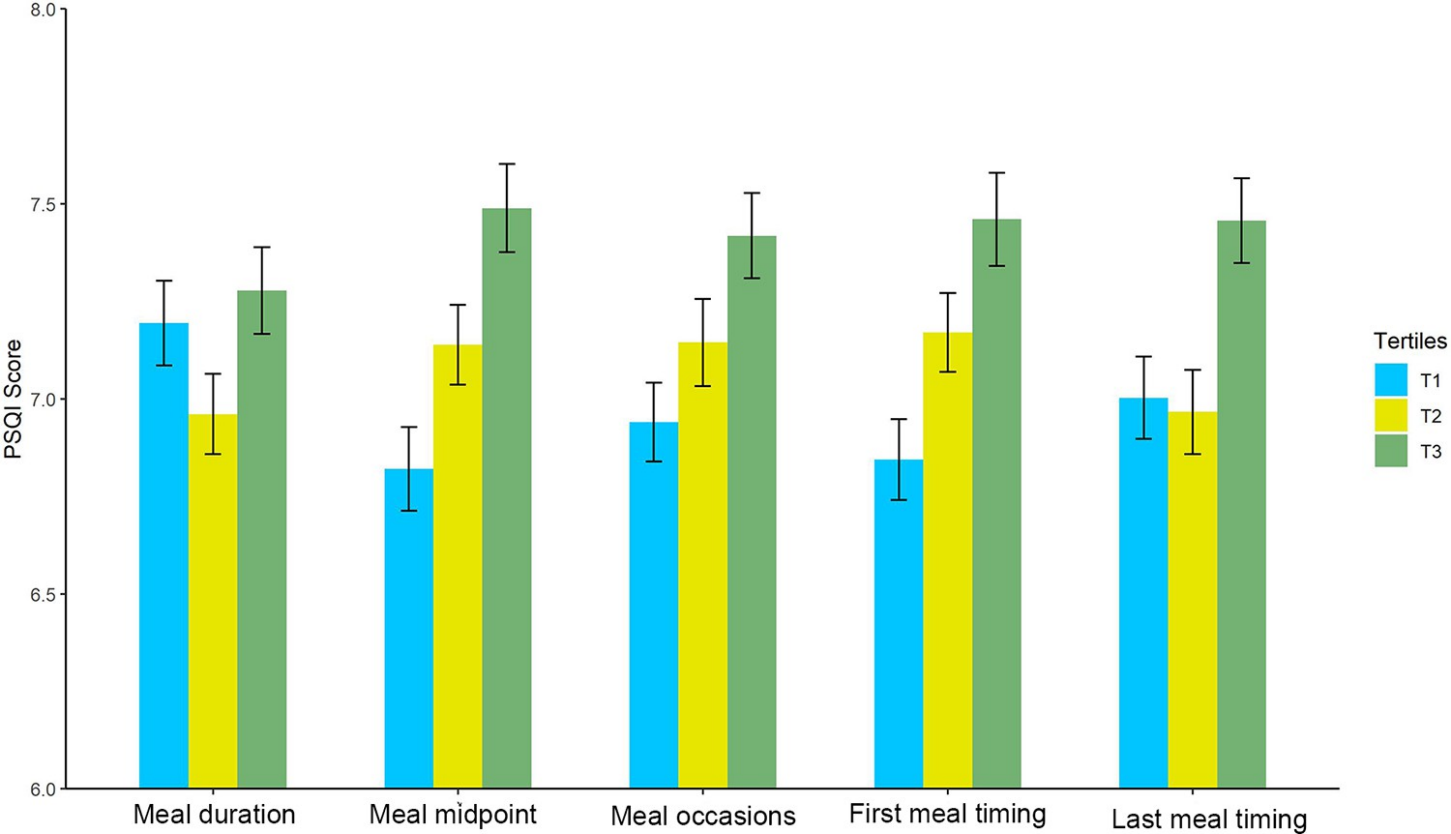
The relationship between the three-category variables of meal timing, meal durations, meal occasions, and sleep quality.

Fig 1 illustrates the relationship between the three-category variables of meal timing, meal durations, meal occasions, and sleep quality using bar graphs and error bars. We refined our categorization approach, segregating the population into three distinct groups based on individual parameters: first meal timing, meal midpoint, last meal timing, meal duration, and meal occasions. The results indicated that, compared to participants in the first tertile of meal occasions and first, midpoint, and last meal timings, those in the last tertile of these factors had higher PSQI scores.

### Relationships between meal timing and PSQI score

Table 2 presents the relationship between meal timing and PSQI scores. Across all models, later meal timings and more meal occasions are associated with higher PSQI scores. No significant associations were found between meal durations and PSQI scores. After adjusting for all covariates, compared to the first tertile, the β (95% CI) of the third tertile is 0.545 (0.226, 0.864) for first meal timing, 0.586 (0.277, 0.896) for midpoint meal timing, and 0.385 (0.090, 0.680) for last meal timing, respectively. Additionally, more meal occasions are associated with higher PSQI scores. After adjusting for all covariates, compared to the first tertile of meal occasions, the β (95% CI) of the third tertile is 0.332 (0.021, 0.642).

**Table 2:** Association between Meal timing and PSQI score.

	Crude model		Model 1		Model 2	
	β(95%CI)	*P*	β(95%CI)	*P*	β(95%CI)	*P*
**First meal timing**						
T1 (Mean 06:52) (n = 2394)	Reference		Reference		Reference	
T2 (Mean 08:15) (n = 2613)	0.326(0.038,0.614)	0.027	0.271(-0.014,0.557)	0.063	0.293(0.009,0.578)	0.043
T3 (Mean 10:00) (n = 2016)	0.616(0.308,0.924)	<0.001	0.513(0.194,0.832)	0.002	0.545(0.226,0.864)	0.001
**Meal midpoint**						
T1 (Mean 13:07) (n = 3353)	Reference		Reference		Reference	
T2 (Mean 14:15) (n = 2448)	0.318(0.024,0.612)	0.034	0.261(-0.030,0.551)	0.078	0.248(-0.042,0.538)	0.093
T3 (Mean 15:30) (n = 2222)	0.669(0.367,0.970)	<0.001	0.631(0.322,0.940)	<0.001	0.586(0.277,0.896)	<0.001
**Last meal timing**						
T1 (Mean 18:45) (n = 2512)	Reference		Reference		Reference	
T2 (Mean 20:15) (n = 2174)	-0.036(-0.335,0.262)	0.811	0.005(-0.29,0.3)	0.974	-0.033(-0.327,0.262)	0.829
T3 (Mean 21:47) (n = 2337)	0.454(0.161,0.747)	0.002	0.448(0.156,0.741)	0.003	0.385(0.090,0.680)	0.011
**Meal occasions**						
T1 (Mean 3.5) (n = 2806)	Reference		Reference		Reference	
T2 (Mean 4.5) (n = 2168)	0.204(-0.087,0.496)	0.169	0.157(-0.131,0.445)	0.285	0.129(-0.161,0.42)	0.384
T3 (Mean 6.0) (n = 2049)	0.478(0.182,0.774)	0.002	0.381(0.083,0.679)	0.012	0.332(0.021,0.642)	0.036
**Meal duration**						
T1 (10.0) (n = 2440)	Reference		Reference		Reference	
T2 (12.0) (n = 2350)	-0.233(-0.528,0.061)	0.120	-0.169(-0.46,0.122)	0.255	-0.220(-0.512,0.072)	0.140
T3 (14.1) (n = 2233)	0.083(-0.215,0.382)	0.584	0.183(-0.114,0.480)	0.228	0.039(-0.263,0.341)	0.801

Crude model adjusts for: None

Model 1 adjusts for: Age, Sex, Race, Education, Poverty ratio, and Marital;

Model 2 adjusts for: Age, Sex, Race, Education, Poverty ratio; Marital, BMI, Smoke; Drinking, healthy eating index 2015, and total energy intake.

## Discussion

This study utilized data from NHANES 2005 to 2008 to characterize the chrono-nutrition patterns of the population and investigate their association with sleep quality, as measured by the PSQI. Our findings indicated that later meal timings, including the first meal timing, meal midpoint, and last meal timing, as well as more meal occasions, were associated with higher PSQI scores.

In the chrono-nutrition study, later meal timings, including first, midpoint, and last meal timings, were associated with poorer sleep quality. A study demonstrated that the temporal characteristics of meal patterns may affect various aspects of sleep health [24, 25]. Both short and long sleep durations with poor sleep quality have been reported to be related to irregular meal patterns in middle-aged and older adults [26], and irregular eating habits were associated with sleep loss [27]. Xian et al. reported that regular breakfast consumption increased morning and intermediate sleep chronotypes and improved sleep quality in college students [28]. Chung N et al reported that adjustments in meal timing can influence sleep patterns and overall sleep quality [29, 30]. Furthermore, a chronobiological study reported that the most significant risk factors for the development of carbohydrate metabolism disorders, accompanied by circadian dysfunction in the surveyed population, include irrational distribution of the energy value of food throughout the day, frequent meals, late breakfast and dinner, shifting bedtime, shortening sleep duration, and exposure to artificial lighting in the evening [31]. This evidence primarily demonstrated that irregular meal timing diminished sleep quality. Loo et al. reported that poor sleep quality was associated with later meals among pregnant women [32]. In this study, we provided additional evidence on the relationship between meal timing and sleep quality. On the one hand, eating later and sleeping later were more likely to coincide, leading to irregular sleep/wake cycles. On the other hand, the relationship between later meal timing and eating regularity should be further studied to elucidate the connection between meal timing and sleep quality.

In addition, the timing of meals can potentially influence the quality of sleep via a multitude of mechanisms. For example, the dynamic interaction among circadian rhythms, sleep patterns, and behavioral aspects is vital in the maintenance of health. Alterations in dinner timings can lead to modifications in sleep patterns, thereby potentially influencing the circadian rhythm. Meal timing, serving as a critical cue for peripheral biological clocks, can modulate sleep quality through the regulation of the central biological clock’s rhythm [33]. Studies have also demonstrated that meal timing exerts an effect on the circadian rhythm via post-transcriptional modifications, entailing alterations in the phosphorylation of certain proteins [34]. Therefore, meal timing has the potential to regulate metabolism, and circadian rhythms. A diminished time gap between last meal and bedtime can result in a prolonged sleep latency period, indicating a potential correlation between the timing of meals and challenges in initiating sleep [35]. Hormones that demonstrate circadian rhythms, such as insulin, leptin, and ghrelin, regulate the digestive and absorptive processes. Consequently, these hormones might exert an influence on the hypothalamic region of the brain, which in turn impacts sleep quality [36]. Additionally, individuals consuming their evening meal at later times might encounter reduced sleep quality, which is potentially linked to digestive system disorders [37].

Our study has some strengths. Firstly, we used a nationally representative data with a large sample size from NHANES. Secondly, a chrono-nutrition approach was used to explore the association of meal timing and meal occasions with sleep quality, which provided new evidence of dietary behavior and sleep quality. It is important to recognize certain limitations inherent in our study. Firstly, given the cross-sectional nature of our study, it precludes the establishment of a causal relationship between meal timing and PSQI. Secondly, despite adjusting for potential confounding factors, the presence of residual or unaccounted confounding effects attributable to measurement errors or unmeasured variables cannot be ruled out. For instance, physical activity, which also significantly influences sleep quality, was not included as a covariate in our analysis. Further research should consider including such factors to provide a more comprehensive understanding of the influences on sleep quality. Finally, caution must be exercised in generalizing the study’s conclusions to other age groups or geographical contexts, given that our research was confined to U.S. adults aged 18 and over.

## Conclusion

While various factors influence sleep quality, our research, grounded in chronobiology, reveals a correlation between later meal timing and higher PSQI scores, indicating poorer sleep quality. These findings offer valuable insights into the realm of chronobiology and underscore the criticality of incorporating meal timing considerations in sleep and health-related research endeavors. However, the specific mechanisms and causal relationships necessitate further experimental elucidation.

## Supporting information

S1 Table Healthy eating index-2015 components & scoring standards. (DOCX)
