# Supplementary material for: Chronobiological perspectives: Association between meal timing and sleep quality
Source: PLoS One. 2024 Aug 1;19(8):e0308172. doi: 10.1371/journal.pone.0308172 (PMC11293727; doi:10.1371/journal.pone.0308172)
Supplement: S1 Table — (DOCX) [file pone.0308172.s001.docx]

**Table S1. Healthy Eating Index-2015 Components & Scoring Standards**

| Component | Maximum points | Standard for maximum score | Standard for minimum score of zero |  |
| --- | --- | --- | --- | --- |
| *Adequacy* | | | | |
| Total Fruits[^2^](https://epi.grants.cancer.gov/hei/developing.html#f2) | 5 | ≥0.8 cup equiv. per 1,000 kcal | No Fruit |  |
| Whole Fruits[^3^](https://epi.grants.cancer.gov/hei/developing.html#f3) | 5 | ≥0.4 cup equiv. per 1,000 kcal | No Whole Fruit |  |
| Total Vegetables[^4^](https://epi.grants.cancer.gov/hei/developing.html#f4) | 5 | ≥1.1 cup equiv. per 1,000 kcal | No Vegetables |  |
| Greens and Beans[^4^](https://epi.grants.cancer.gov/hei/developing.html#f4) | 5 | ≥0.2 cup equiv. per 1,000 kcal | No Dark Green Vegetables or Legumes |  |
| Whole Grains | 10 | ≥1.5 oz equiv. per 1,000 kcal | No Whole Grains |  |
| Dairy[^5^](https://epi.grants.cancer.gov/hei/developing.html#f5) | 10 | ≥1.3 cup equiv. per 1,000 kcal | No Dairy |  |
| Total Protein Foods[^6^](https://epi.grants.cancer.gov/hei/developing.html#f6) | 5 | ≥2.5 oz equiv. per 1,000 kcal | No Protein Foods |  |
| Seafood and Plant Proteins[^6^](https://epi.grants.cancer.gov/hei/developing.html#f6)^,^[^7^](https://epi.grants.cancer.gov/hei/developing.html#f7) | 5 | ≥0.8 oz equiv. per 1,000 kcal | No Seafood or Plant Proteins |  |
| Fatty Acids[^8^](https://epi.grants.cancer.gov/hei/developing.html#f8) | 10 | (PUFAs + MUFAs)/SFAs ≥2.5 | (PUFAs + MUFAs)/SFAs ≤1.2 |  |
| *Moderation* | | | | |
| Refined Grains | 10 | ≤1.8 oz equiv. per 1,000 kcal | ≥4.3 oz equiv. per 1,000 kcal |  |
| Sodium | 10 | ≤1.1 gram per 1,000 kcal | ≥2.0 grams per 1,000 kcal |  |
| Added Sugars | 10 | ≤6.5% of energy | ≥26% of energy |  |
| Saturated Fats | 10 | ≤8% of energy | ≥16% of energy |  |

**(1)** Intakes between the minimum and maximum standards are scored proportionately.

**(2)** Includes 100% fruit juice.

**(3)** Includes all forms except juice.

**(4)** Includes legumes (beans and peas).

**(5)** Includes all milk products, such as fluid milk, yogurt, and cheese, and fortified soy beverages.

**(6)** Includes legumes (beans and peas).

**(7)** Includes seafood, nuts, seeds, soy products (other than beverages), and legumes (beans and peas).

**(8)** Ratio of poly- and monounsaturated fatty acids (PUFAs and MUFAs) to saturated fatty acids (SFAs).

*Adequacy components* represent the food groups, subgroups, and dietary elements that are encouraged. For these components, higher scores reflect higher intakes, because higher intakes are desirable.

*Moderation components* represent the food groups and dietary elements for which there are recommended limits to consumption. For moderation components, higher scores reflect lower intakes, because lower intakes are more desirable.

**Reference**

1. Krebs-Smith SM, Pannucci TE, Subar AF, et al. Update of the Healthy Eating Index: HEI-2015. J Acad Nutr Diet. Sep 2018;118(9):1591-1602.

2. National Cancer Institute. HEI Scoring Algorithm. Accessed August, 2022. https://epi.grants.cancer.gov/hei/hei-scoring-method.html
